# Effects of dichloroacetate as single agent or in combination with GW6471 and metformin in paraganglioma cells

**DOI:** 10.1038/s41598-018-31797-5

**Published:** 2018-09-11

**Authors:** Rosalba Florio, Laura De Lellis, Serena Veschi, Fabio Verginelli, Viviana di Giacomo, Marialucia Gallorini, Silvia Perconti, Mario Sanna, Renato Mariani-Costantini, Angelica Natale, Arduino Arduini, Rosa Amoroso, Amelia Cataldi, Alessandro Cama

**Affiliations:** 1 Department of Pharmacy, “G. d’Annunzio” University of Chieti-Pescara, Chieti, Italy; 2 Unit of General Pathology, CeSI-MeT, University of Chieti, Chieti, Italy; 3 Department of Medical, Oral and Biotechnological Sciences, “G. d’Annunzio” University of Chieti-Pescara, Chieti, Italy; 4 Department of Otology and Skull Base Surgery, Gruppo Otologico, Piacenza, Italy; 5 R&D Department, CoreQuest Sagl, Manno, Switzerland

## Abstract

Paragangliomas (PGLs) are infiltrating autonomic nervous system tumors that cause important morbidity. At present, surgery is the only effective therapeutic option for this rare tumor. Thus, new agents for PGL treatment should be identified. Using unique PGL cell models established in our laboratory, we evaluated the effect of dichloroacetate (DCA) as single agent or in a novel combination with other metabolic drugs, including GW6471 and metformin. DCA and metformin had not been tested before in PGL. DCA reduced PGL cell viability and growth through mechanisms involving reactivation of PDH complex leading to promotion of oxidative metabolism, with lowering of lactate and enhanced ROS production. This resulted in cell cycle inhibition and induction of apoptosis in PGL cells, as shown by flow cytometry and immunoblot analyses. Moreover, DCA drastically impaired clonogenic activity and migration of PGL cells. Also metformin reduced PGL cell viability as single agent and the combinations of DCA, GW6471 and metformin had strong effects on cell viability. Furthermore, combined treatments had drastic and synergistic effects on clonogenic ability. In conclusion, DCA, GW6471 and metformin as single agents and in combination appear to have promising antitumor effects in unique cell models of PGL.

## Introduction

Paragangliomas (PGLs) are weakly metastatic, but highly infiltrating tumors that arise in sympathetic and parasympathetic paraganglia^1^. Approximately 80–85% of these tumors develop from the adrenal medulla and are indicated as pheochromocytomas (PCCs), whereas 15–20% are in extra-adrenal chromaffin tissue and are named secreting paragangliomas (sPGLs)^1^. The term paraganglioma is also used to describe head and neck tumors derived from parasympathetic tissue. PGLs may arise from hereditary predisposition (over 30% of PGL cases), with germline mutations in the genes encoding for mitochondrial complex II (succinate dehydrogenase, SDH) subunits (*SDHA, SDHB, SDHC, SDHD*) or cofactors, including *SDHAF2* that is responsible for the flavination of the SDHA subunit^2^. SDH is a mitochondrial complex that participates in both Krebs cycle and electron transport chain^3,4^. Head and neck PGLs may remain clinically silent for years, due to their slow growth, but they can induce manifestations related to the infiltration of the adjacent neurovascular structures and of the skull base^2^. At present, surgery is the only effective therapeutic option for head and neck PGL^5^. When surgical eradication is not achievable, radiotherapy and chemotherapy may be used, but only partial responses are observed^6^. Hence, novel therapeutic agents that could be employed in PGL treatment are urgently needed. However, this task is largely unexplored in PGL, also due to the lack of commercially available cell lines for this rare tumor.

Tumor metabolism is considered a valuable target for antitumor therapy and molecules active on metabolism, including those modulating nuclear receptors, show promising antitumor effects in different cancer models^7^. We recently established unique *in vitro* models of head and neck PGL and showed that the specific PPARα antagonist GW6471 reduced cell viability, interfered with cell cycle, induced caspase-dependent apoptosis and markedly impaired clonogenicity in head and neck PGL cells, supporting PPARα inhibition as a novel therapeutic target for this chemoresistant tumor^8,9^.

Among drugs active on tumor metabolism, dichloroacetate (DCA) is a structural analog of pyruvate that inhibits pyruvate dehydrogenase kinase (PDK) stimulating pyruvate dehydrogenase (PDH) activation. This has been reported to reverse tumor-associated increase in glycolysis (Warburg effect), leading to a decreased cancer malignancy^10^. By blocking PDK, DCA decreases lactate production switching the metabolism of pyruvate from glycolysis towards oxidative phosphorylation in the mitochondria and this property has been exploited in the treatment of lactic acid accumulation disorders^11^. In addition, several *in vitro* and/or *in vivo* studies have shown that DCA is able to suppress cancer cells via inhibition of PDK by inducing apoptosis and/or by interfering with cell cycle and proliferation in many tumors, including pancreatic, breast, endometrial and ovarian cancers, neuroblastoma and T-cell lymphomas^12–20^. Improved antitumor effects were also reported by combining DCA with radiation or other drugs^10^. Based on these encouraging results, several clinical trials have been developed to test the antitumor effects of DCA, when used alone or in combination, in different human cancers^21,22^.

The effects of DCA were never tested in PGLs and, based on the above-mentioned considerations, in the present study we evaluated its antitumor potential in PGL cell lines established from this rare tumor in our laboratory. We further tested the effect of a novel combination among DCA and other metabolic agents on PGL cells. In particular, we combined DCA with the specific PPARα antagonist GW6471, which we had previously shown to be effective in PGL cell lines^9^, and metformin, not tested before in PGL. The combination of these three metabolic drugs has not been tested before in any tumor cell line. Metformin is a biguanide derivative widely used as anti-hyperglycemic drug, which has been investigated for its anticancer effects, as it was shown to reduce cancer risk^23^. Metformin displayed antitumor activity in breast, prostate, lung, colon cancers and gliomas^24–28^, targeting several crucial metabolic processes and signaling pathways in cancer cells^29^. Previous studies showed that metformin had synergistic antitumor effect when used in combination with DCA in several tumors, including breast, ovarian and lung cancers^30–32^.

Our results show that DCA reduces PGL cell viability through mechanisms involving PDK inhibition, resulting in reactivation of PDH complex that leads to promotion of oxidative metabolism, lowering of lactate production and enhancing of intracellular production of reactive oxygen species (ROS). This ultimately promotes both cell cycle arrest and apoptosis in PGL cells. We also show that metformin affects PGL cell viability as single agent and that the combination of DCA, metformin and GW6471 has a profound impact on PGL cell viability and clonogenicity. Thus, DCA, metformin and GW6471, when used alone or in combination, represent promising antitumor agents for this rare tumor poorly responsive to chemotherapy.

## Results

### Effects of DCA on viability and growth of PGL cells

**Figure 1:**
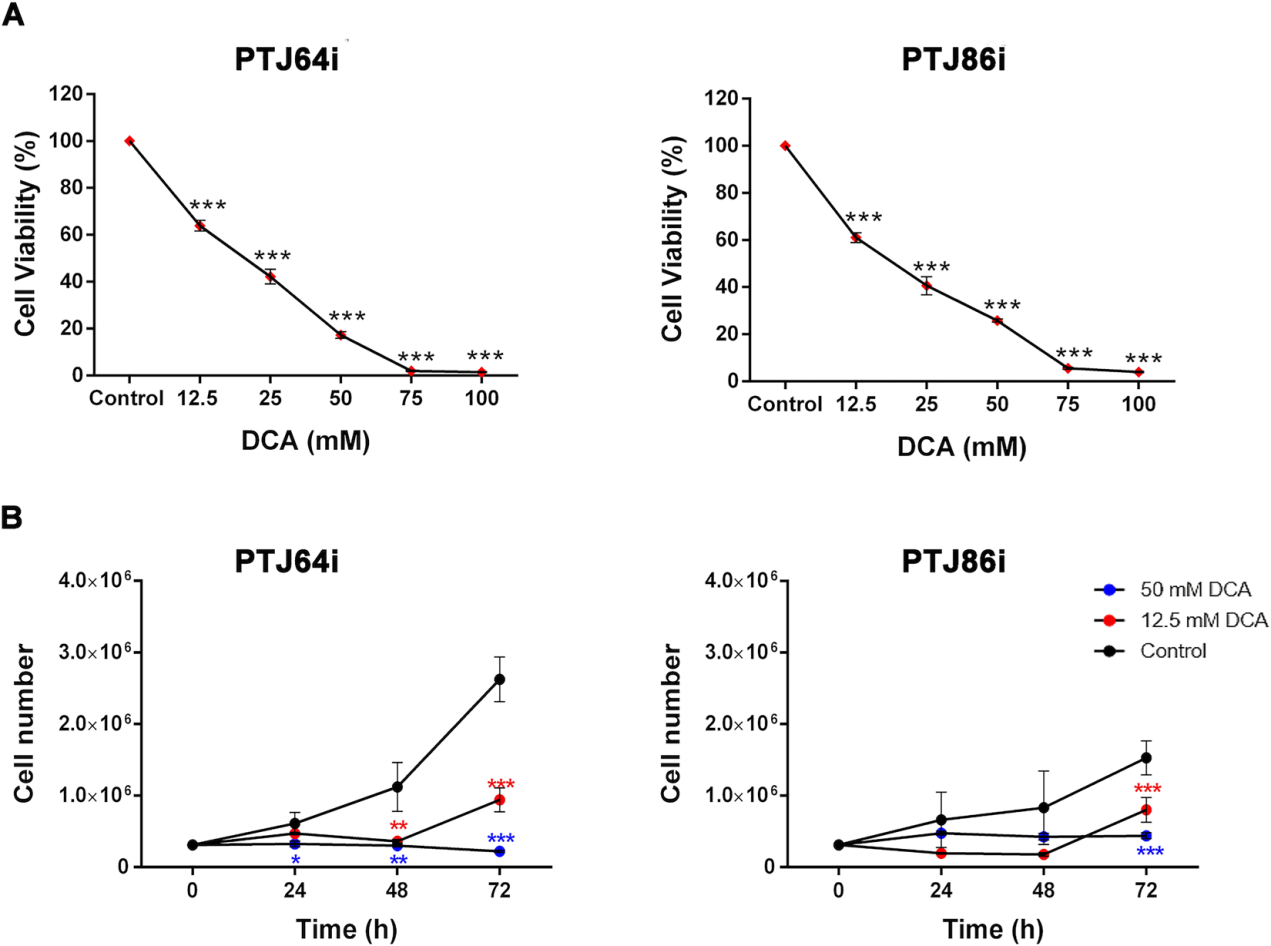
DCA reduces cell viability and growth in PTJ64i and PTJ86i cell lines. **(A)** Cells were incubated for 72 hours with DCA at the indicated concentrations, or with PBS vehicle (control). Cell viability was assessed by MTT assay. DCA significantly inhibited cell viability in both cell lines in a dose–dependent manner, with an IC_50_ of 18.9 mM in PTJ64i and 18.8 mM in PTJ86i, as assessed by CompuSyn. Data shown are the means ± SD of three independent experiments with quintuplicate determinations. *Statistically significant differences between control and each drug concentration (***p < 0.001); **(B)** Cell number was measured over a 72–hour time course treatment with 12.5 mM, 50 mM DCA or with vehicle control. Data shown are the means ± SD of three to nine independent determinations (*p < 0.05; **p < 0.01; ***p < 0.001).

To determine the effect of DCA on the viability of head and neck PGL cells, we incubated PTJ64i and PTJ86i cell lines for 72 hours with the indicated concentrations of the drug. Treatment with DCA significantly reduced cell viability in a dose-dependent manner in both PTJ64i and PTJ86i cell lines (Fig. 1, panel A), with IC50s comparable to those previously observed in other tumor cell lines^33,34^. We next investigated the effect of DCA on PTJ64i and PTJ86i cell growth (Fig. 1, panel B). Treatment with DCA at 12.5 or 50 mM markedly and significantly inhibited cell growth of PTJ64i at 48 and 72 hours as compared to vehicle, while in PTJ86i the effect of DCA on cell growth was evident only at 72 hours (Fig. 1, panel B).

### DCA inhibits PDK, reduces extracellular lactate levels and enhances ROS production in PGL cells

**Figure 2:**
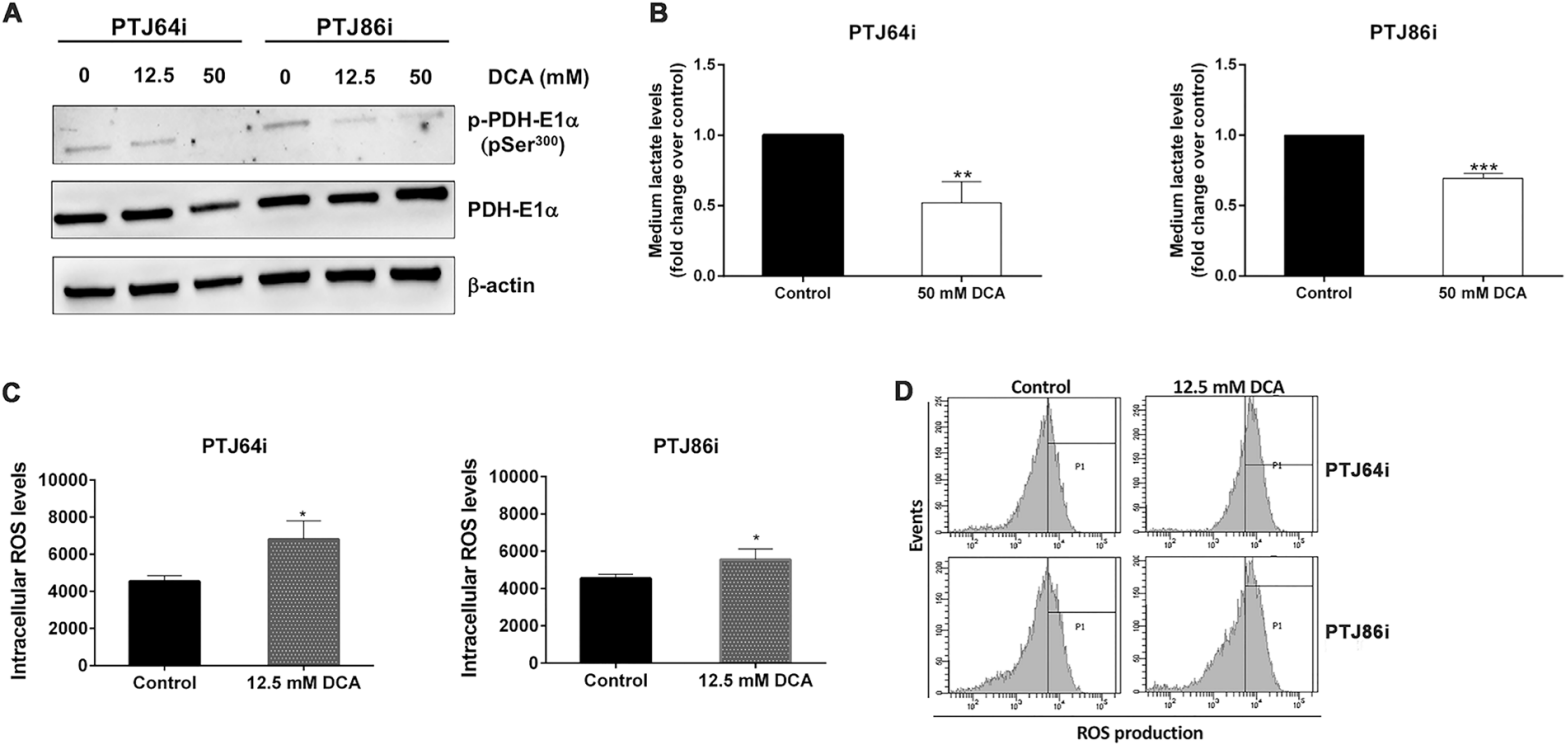
DCA inhibits PDK, decreases extracellular lactate and increases intracellular ROS levels in PTJ64i and PTJ86i cell lines. **(A)** Western blot analysis of p-PDH-E1α (pSer^300^) and PDH-E1α proteins after incubation of PGL cells for 24 hours with vehicle or DCA at the indicated concentrations; **(B)** Histograms represent quantification of extracellular lactate levels in the culture media of PGL cell lines treated with DCA or vehicle (control) for 24 hours. Data shown are the means ± SD of three replicates and are expressed as fold change relative to vehicle (control) (**p < 0.01; ***p < 0.001); **(C)** Intracellular ROS levels in PTJ64i and PTJ86i cells measured by flow cytometry after a 24–hour treatment with DCA or vehicle (control). Data shown are the means ± SD of three independent experiments (*p < 0.05); **(D)** Representative CM–H2DCFDA histograms derived from flow cytometry analysis showing ROS production in PTJ64i and PTJ86i cells after a 24–hour treatment with DCA or vehicle (control). Full–length western blots are included in Supplementary Information (Fig. S1).

A mechanism by which DCA may affect cell viability is through inhibition of PDK and increased ROS production. In this regard, DCA was previously shown to inhibit PDK in different tumor cell lines, resulting in dephosphorylation and reactivation of the PDH complex^33^, that promotes oxidative phosphorylation leading to increased ROS production^35–37^. To evaluate whether DCA treatment induced dephosphorylation of PDH in PGL cells, we analyzed by western blotting the phosphorylation status of the PDH-E1α  subunit. DCA treatment reduced phosphorylation of PDH-E1α (pSer^300^), without affecting PDH-E1α expression, in both PGL cell lines (Fig. 2, panel A), confirming that DCA inhibits the expected target PDK, thus activating the PDH complex in PGL cells. As PDH activation is expected to promote oxidative metabolism of pyruvate at the expense of lactate production, we measured lactate levels in growth media of PGL cells treated with DCA or vehicle. A 24-hour treatment of cells with DCA induced a marked and significant reduction of extracellular lactate levels in the culture media of both PGL cell lines (Fig. 2, panel B). Considering that activation of oxidative metabolism is commonly associated to increased ROS production, we analyzed whether DCA treatment affected ROS generation in PGL cells. A 24-hour treatment with DCA significantly increased ROS production in both PGL cell lines as compared to the vehicle control (Fig. 2, panels C and D). The increase appeared more pronounced in PTJ64i as compared to PTJ86i cells (Fig. 2, panel C). These findings show that DCA treatment promotes oxidative metabolism through PDK inhibition, thus increasing ROS levels in PGL cells.

### DCA affects cell cycle in PGL cells

**Figure 3:**
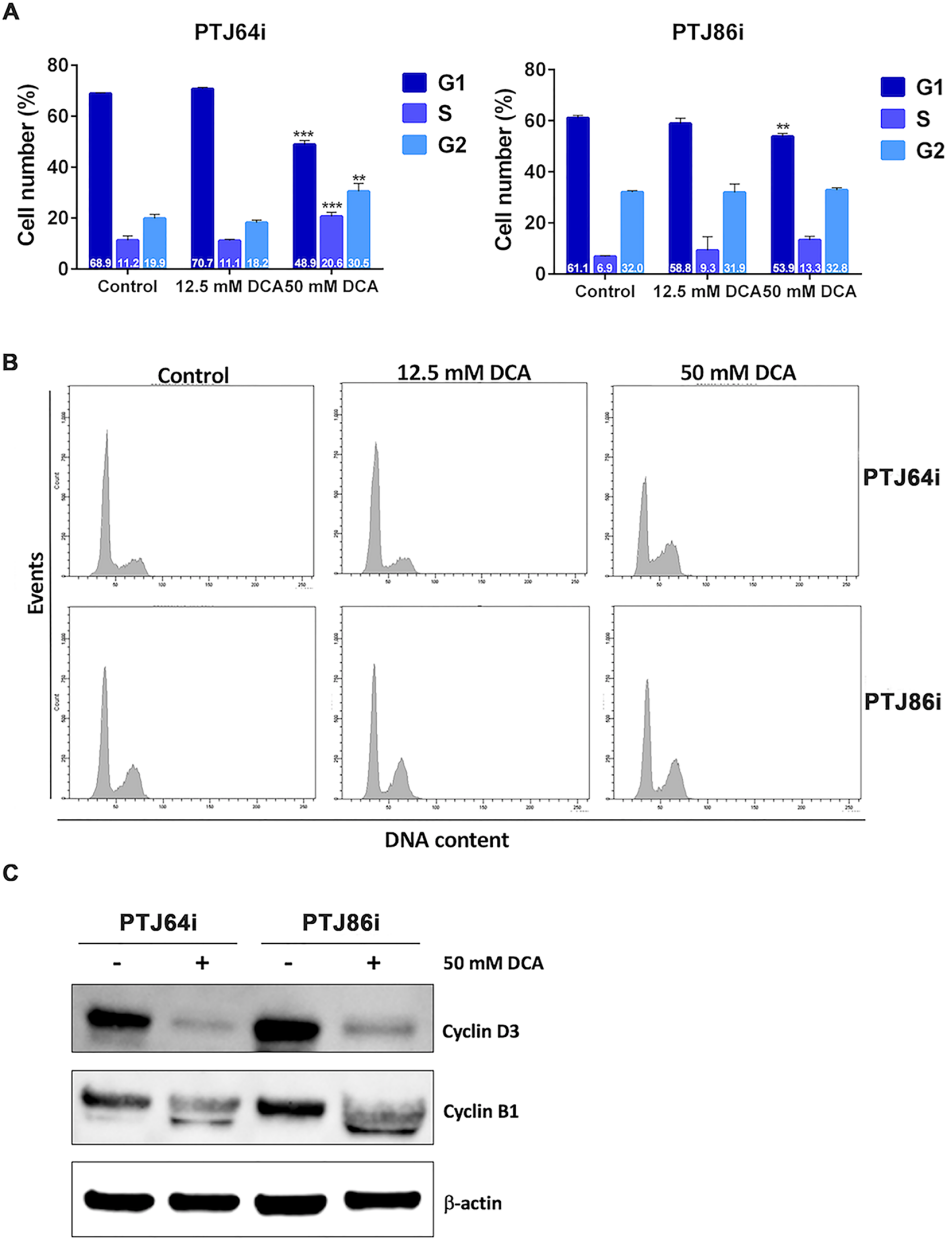
DCA affects cell cycle in PTJ64i and PTJ86i cell lines. **(A)** The histograms show the mean percentages of cells (values inside the bars) in the different cell phases, evaluated by flow cytometry, after 24-hour treatment with 12.5 or 50 mM DCA as compared to control. Results shown are the means ± SD of three independent experiments (**p < 0.01; ***p < 0.001); **(B)** Representative DNA distribution histograms of PGL cells exposed to DCA (12.5 mM and 50 mM) or vehicle (control) for 24 hours, as measured by flow cytometry; **(C)** Expression of cyclin D3 and cyclin B1 proteins after incubation of cells for 24 hours with vehicle or 50 mM DCA was analyzed by western blot using antibodies directed against the indicated proteins. Full–length western blots are included in Supplementary Information (Fig. S2).

Considering that DCA treatment increased ROS production, which is known to affect cell cycle regulation^38^, we explored whether altered cell cycle progression could contribute to the decreased PGL cell viability. Cell cycle was evaluated by flow cytometry in both cell lines treated with DCA at 12.5 mM, 50 mM or vehicle for 24 hours. At 12.5 mM DCA, PTJ64i and PTJ86i cells showed similar cell cycle distributions as compared to vehicle (Fig. 3, panels A and B), whereas at 50 mM DCA affected cell cycle in both cell lines. In particular, 50 mM DCA significantly decreased the percentage of PTJ64i cells in G1 phase as compared to untreated cells (from 68.9% to 48.9%; *p* < 0.001) and increased the percentage of cells in S phase (from 11.2% to 20.6%; *p* < 0.01) as well as in G2/M phase (from 19.9% to 30.5%; *p* < 0.01). In PTJ86i, treatment with 50 mM DCA significantly decreased the percentage of cells in G1 phase as compared to untreated cells (from 61.1% to 53.9%; *p* < 0.01) and increased the percentage of cells in S phase (from 6.9% to 13.3%; *p* < 0.01). To investigate the mechanism of cell cycle inhibition observed with 50 mM DCA, we evaluated the expression of cell cycle-related proteins by western blot in response to this treatment. Both cell lines were treated for 24 hours with 50 mM DCA and then the cell lysates were immunoblotted with antibodies against cyclin B1 and cyclin D3. In PTJ64i and PTJ86i the expression of cyclin D3 was drastically reduced after treatment and a decreased expression of cyclin B1 was observed in PTJ64i. These findings were in line with the cell cycle perturbations observed by flow cytometry (Fig. 3, panel C). Therefore, the decreased viability observed by MTT after DCA treatment in both PGL cell lines is related at least in part to cell cycle inhibition.

### DCA induces apoptosis in PGL cells

**Figure 4:**
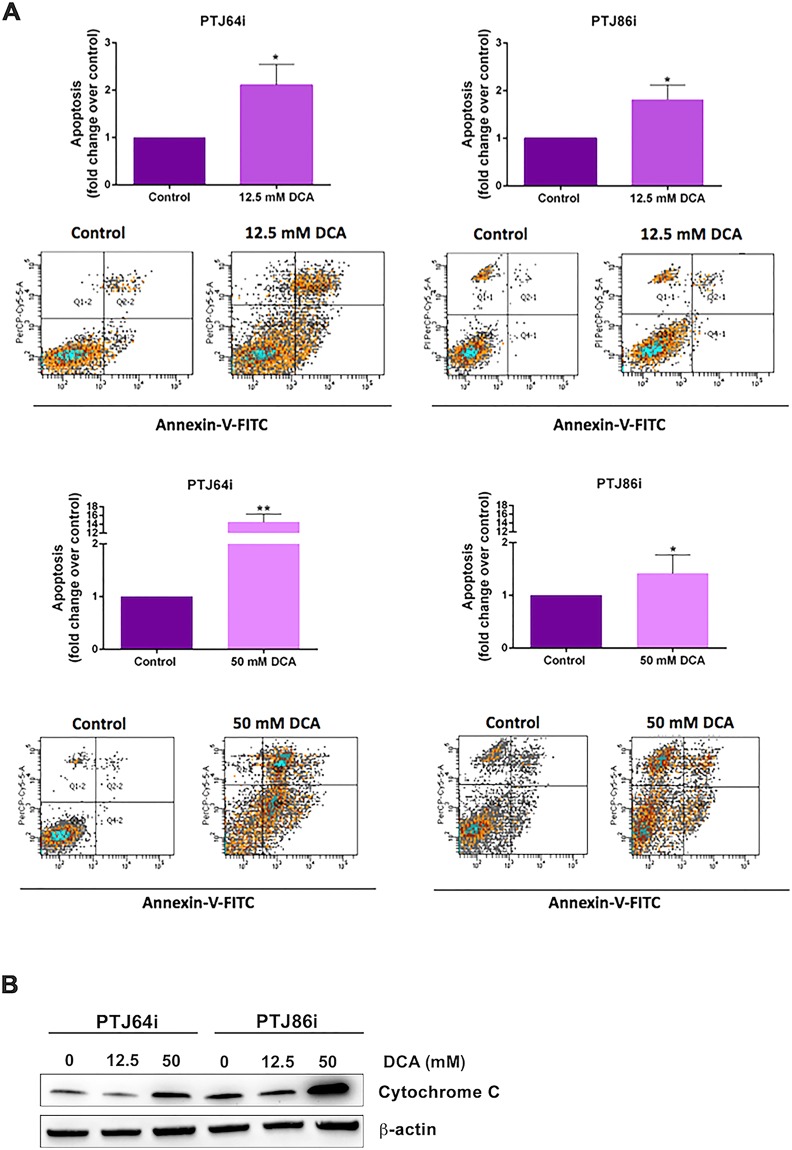
Apoptosis in PTJ64i and PTJ86i cells treated with 12.5 or 50 mM DCA. **(A)** Values represented in the histograms (*top of each panel*) are the means ± SD of two to six independent experiments (*p < 0.05; ***p < 0.001). Dot plots (*bottom of each panel*) show representative experiments after a 24-hour treatment with 12.5 or 50 mM DCA; **(B)** Expression of cytochrome C in PTJ64i and PTJ86i cells treated with 12.5 and 50 mM DCA for 8 hours was analyzed by western blot. Full-length western blots are included in Supplementary Information (Fig. S3).

As DCA-mediated increase in ROS production is known to induce oxidative cellular damage and apoptosis in breast cancer and myeloma cells^36,39^, we investigated whether treatment of PGL cells with DCA was associated with promotion of apoptosis. As indicated by flow cytometry evaluation of Annexin-V staining, DCA treatment resulted in a significant induction of apoptosis in both cell lines (Fig. 4, panel A). At 12.5 mM DCA, the fold increase in apoptotic cells was comparable for PTJ64i and PTJ86i cells. At 50 mM DCA, PTJ64i showed a sharp increment of apoptotic cells that in PTJ86i was not as marked. In line with DCA-induced apoptosis, cytochrome C, an early marker of the intrinsic pathway of apoptosis and an indicator of mitochondrial damage, showed a marked increase in the expression by western blot analysis at 50 mM DCA in both PGL cell lines as compared to control (Fig. 4, panel B). These results indicate that programmed cell death contributes to the reduced viability associated with DCA in PGL cells.

### DCA inhibits clonogenicity and migration of PGL cells

**Figure 5:**
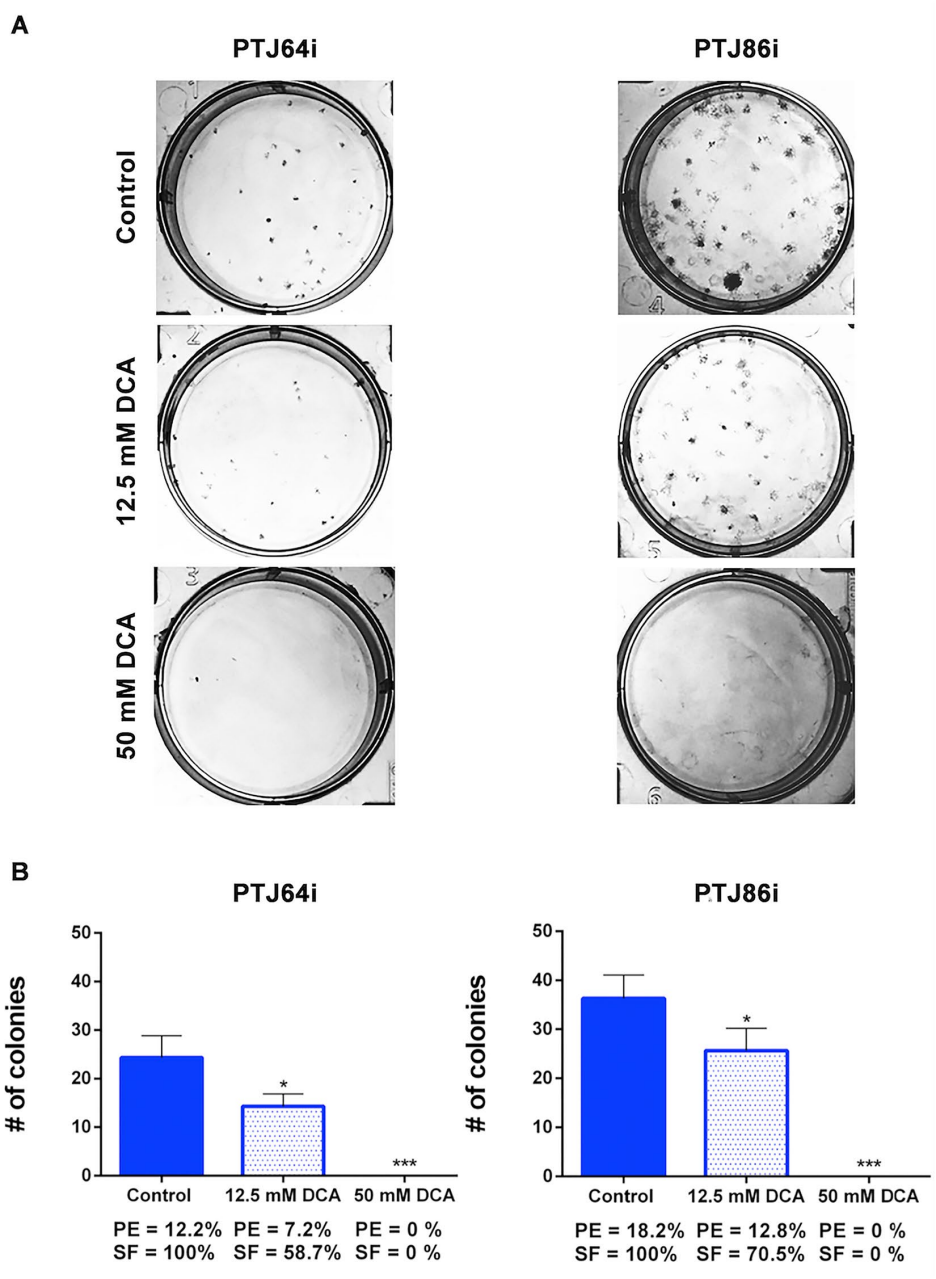
DCA affects clonogenic activity of PTJ64i and PTJ86i cell lines. **(A)** Representative plates of colony formation assays for both PGL cell lines are shown; **(B)** Histograms show the means ± SD of three independent experiments (*p < 0.05; ***p < 0.001). PE: plating efficiency [(# of colonies formed/# of cells plated)*100]; SF: surviving fraction [# of colonies formed *100/(# of cells plated *PE of control vehicle)].

To analyze the effects of DCA on colony formation capacity, we performed a clonogenic assay. Treatment with 12.5 mM DCA significantly reduced clonogenic activity of both cell cultures, as indicated by the reduced plating efficiencies and surviving fractions as compared to vehicle control (Fig. 5, panels A and B). The effects of 50 mM DCA on clonogenic activity were drastic, resulting in no colony formation with both cell lines (Fig. 5, panels A and B).

We further investigated the effects of DCA on PGL cell migration using a wound-healing assay. Confluent monolayers of PTJ64i and PTJ86i cells were treated with vehicle, 12.5 or 50 mM DCA and wound width was evaluated at 0, 24, 48 and 56 hours after treatments.

**Figure 6:**
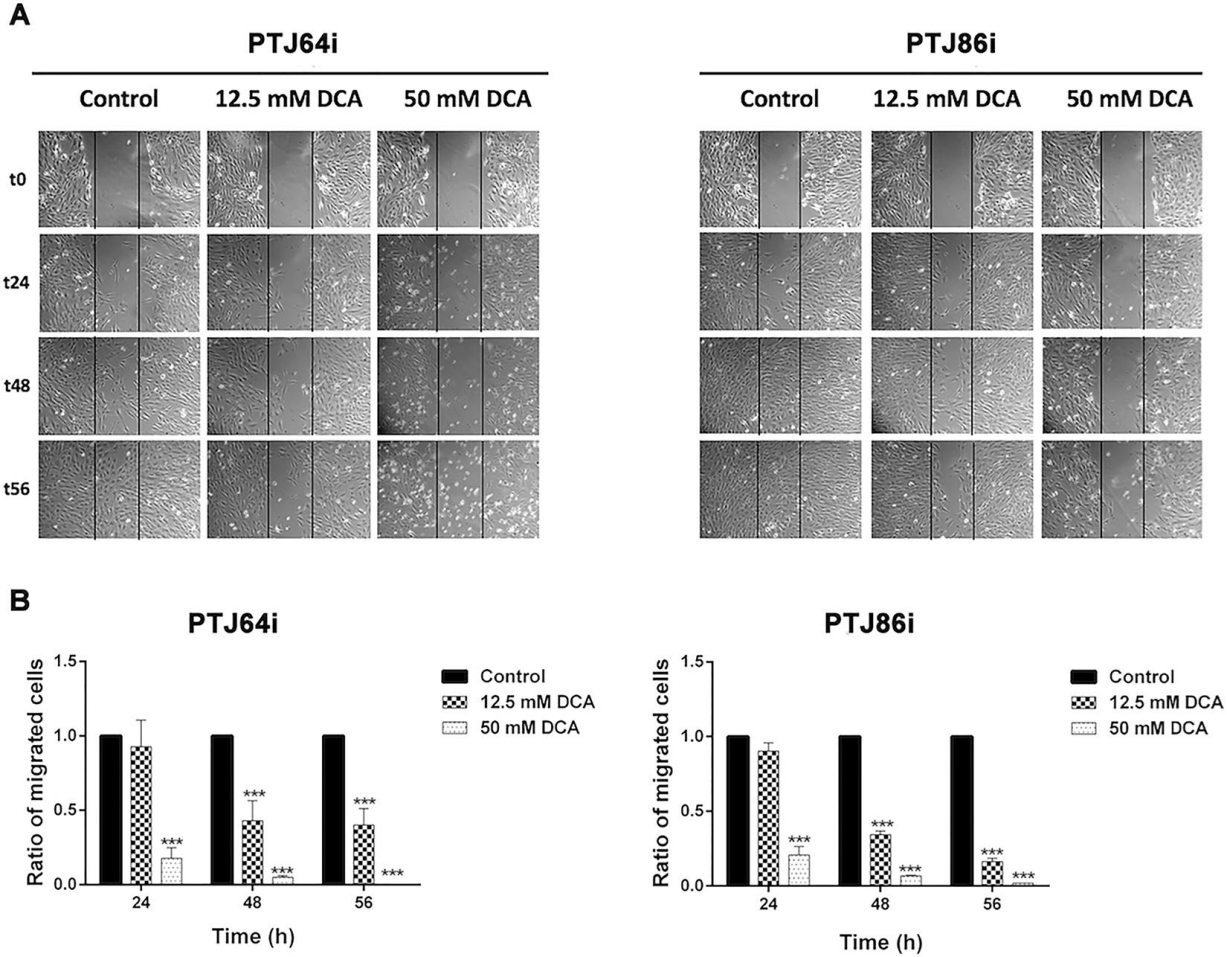
Effect of DCA on wound healing. **(A)** Representative wound-healing assay pictures for PTJ64i and PTJ86i cells treated with vehicle or with DCA (12.5 and 50 mM) are shown. Pictures of PGL cells were taken at 0, 24, 48 and 56 hours to analyze the dynamics of wound closure (vertical lines indicate wound edges); **(B)** Histograms represent quantitative analyses of cell migration and are expressed as the ratio of the number of migrated cells in three fields after treatment as compared with vehicle (***p < 0.001).

At 24 hours, treatment with 12.5 mM DCA had no effect on PGL cell migration (Fig. 6, panels A and B), but there was a marked inhibition of cell migration at later time points. The inhibition of cell migration was more marked at all time points after treatment with 50 mM DCA.

### Effects of combined treatments with DCA, GW6471 and metformin on PGL cell viability

**Figure 7:**
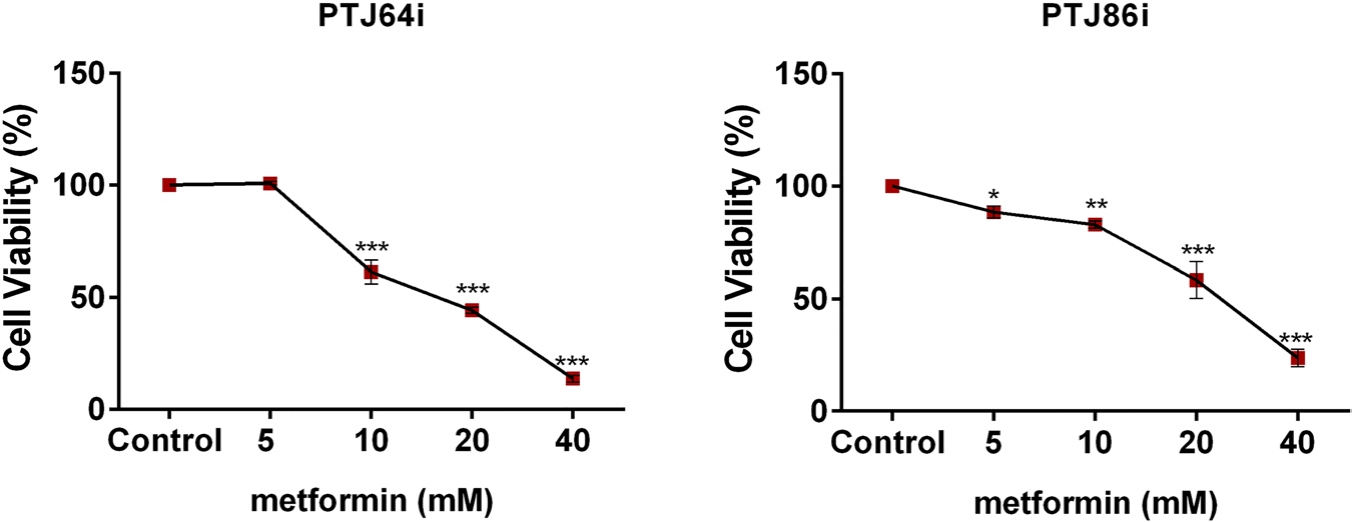
Metformin affects cell viability in PTJ64i and PTJ86i cell lines. Cells were incubated for 72 hours with metformin at the indicated concentrations, or with PBS vehicle (control). Cell viability was assessed by MTT assay. Metformin significantly inhibited cell viability in both cell lines in a dose-dependent manner, with an IC_50_ of 21.6 mM in PTJ64i and 22.1 mM in PTJ86i, as assessed by CompuSyn. Data shown are the means ± SD of three independent experiments with quintuplicate determinations. *Statistically significant differences between control and each drug concentration (*p < 0.05; **p < 0.01; ***p < 0.001).

We studied the effect of combinations including DCA, GW6471 and metformin. Since metformin has never been tested before in PGL, we preliminarily analyzed whether this drug, as single agent, affected viability in PTJ64i and PTJ86i cell lines. As shown in Fig. 7, metformin caused a significant reduction of cell viability in a dose-dependent manner, with IC50s comparable to those previously observed in breast and ovarian tumor cell lines^40–42^.

**Figure 8:**
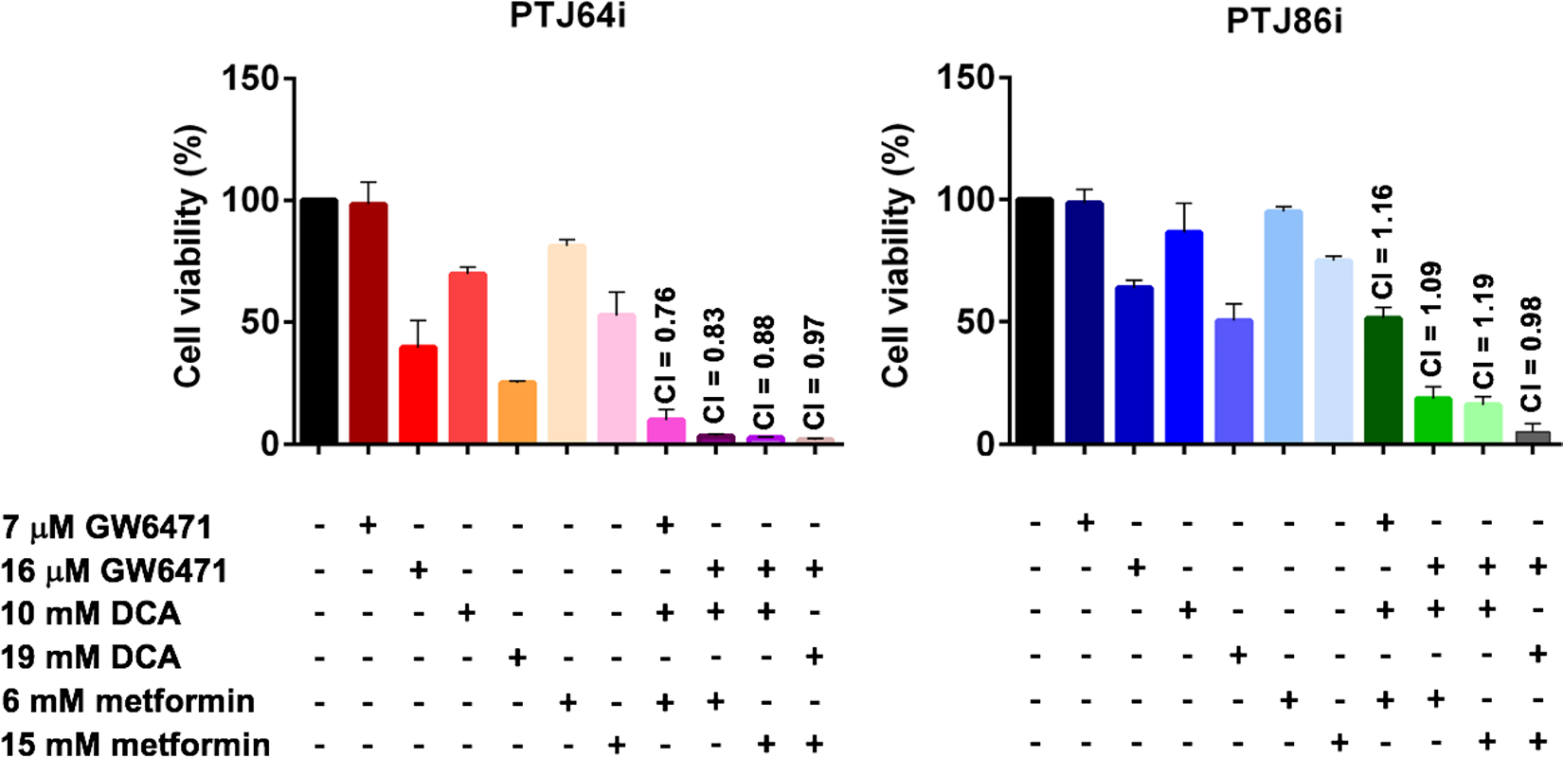
Effect of DCA, GW6471 and metformin as single agents or in combinations on PGL cell viability. Cells were incubated for 72 hours with DCA, GW6471 and metformin, alone or in combination, at the indicated concentrations, or with vehicle. Cell viability was assessed by MTT assay. The combination index (CI) for each drug combination was calculated using the Chou–Talalay equation. Data shown are the means ± SD of three independent experiments with quintuplicate determinations.

To determine the effect of combined treatments with GW6471, DCA and metformin on cell viability we tested two concentrations of each drug as single agent and 4 combinations of these drugs. Overall, the combined treatments caused a greater reduction of cell viability as compared to the effect of single agents at the corresponding concentrations (Fig. 8 and Supplementary Table S1). In PTJ64i the reduction of cell viability was drastic with all combinations tested. In PTJ86i the combination using the lowest doses of each drug had an effect considerably more pronounced as compared to the single agents at the corresponding concentrations. This effect became more marked with combinations using higher drug dosages. Interactions among GW6471, DCA and metformin in the two cell lines were analyzed by CompuSyn software^43^. In PTJ64i all the combined treatments resulted in combination indexes <1, indicating synergistic (CI 0.76, 0.83 and 0.88) or additive (CI 0.97) effects (Fig. 8). In PTJ86i CompuSyn analysis indicated that two drug combinations had additive effects (CI 0.98 and 1.09), whereas two combinations appeared slightly antagonistic (CI 1.16 and 1.19) (Fig. 8).

### Combined treatments with DCA, GW6471 and metformin inhibit clonogenicity

**Figure 9:**
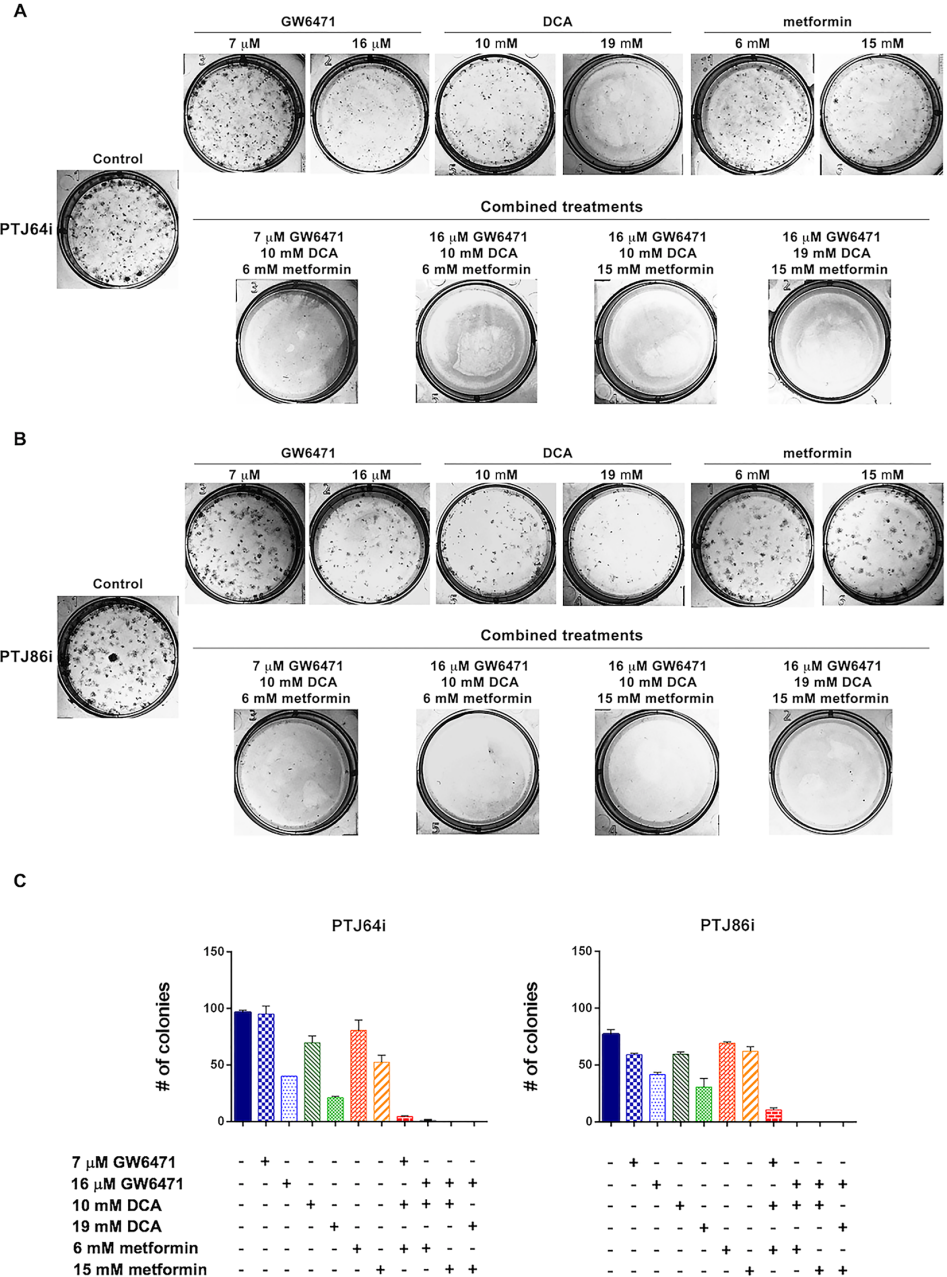
Effect of DCA, GW6471 and metformin as single agents or in combinations on PGL clonogenic capacity. **(A**,**B)** Representative plates of colony formation assays for PTJ64i **(A)** and PTJ86i **(B)** are shown; **(C)** Histograms represent quantitative analyses of colony formation assay in PGL cells. Data shown are the means ± SD of two independent experiments.

The effects of DCA, GW6471 and metformin in combination on the PGL clonogenicity were impressive as compared to each drug used as single agent (Fig. 9 and Supplementary Table S2). Even with the combination using the lowest doses of each drug (7 μM GW6471 + 10 mM DCA + 6 mM metformin) a very marked effect was observed (surviving fractions of 4.69% in PTJ64i and 13.64% in PTJ86i) (Fig. 9 and Table 1). The combination of a higher dose of GW6471 (16 μM) with lower doses of DCA (10 mM) and metformin (6 mM) had a more marked effect than the combination with the lowest doses of each drug, resulting in almost no colony formation in PTJ64i (surviving fraction of 0.52%) or no colony formation in PTJ86i (surviving fraction of 0.00%) (Fig. 9 and Table 1). Notably, all combined treatments employing higher doses of GW6471 (16 μM) and metformin (15 mM), with lower (10 mM) or higher (19 mM) doses of DCA, had a drastic effect on clonogenic activity resulting in no colony formation (Fig. 9 and Table 1). We also evaluated the effects of combined treatments on surviving fractions by CompuSyn and in all combinations the interactions between drugs were synergistic for both cell lines (Table 1). In particular, for PTJ86i the three combinations employing the higher dosage of GW6471 had a very strong synergistic effect (combination indexes < 0.1, Table 1). Thus, all the combinations at lower or higher dosages synergistically reduced self-renewal capacity of PGL cells.

**Table 1:** Plating efficiency (PE), surviving fraction (SF) and combination index (CI) values in PTJ64i and PTJ86i.

	PTJ64i			PTJ86i		
	PE %	SF %	CI	PE %	SF %	CI
Control	48.25	100.00	—	38.50	100.00	—
7 μM GW6471	47.50	98.96	—	29.50	76.62	—
16 μM GW6471	20.00	41.67	—	20.75	53.90	—
10 mM DCA	34.75	72.40	—	29.75	77.27	—
19 mM DCA	10.50	21.88	—	15.25	39.61	—
6 mM metformin	40.25	83.85	—	34.50	89.61	—
15 mM metformin	26.25	54.69	—	31.00	80.52	—
7 μM GW6471 + 10 mM DCA + 6 mM metformin	2.25	4.69	0.65	5.25	13.64	0.40
16 μM GW6471 + 10 mM DCA + 6 mM metformin	0.25	0.52	0.61	0.00	0.00	0.05
16 μM GW6471 + 10 mM DCA + 15 mM metformin	0.00	0.00	0.45	0.00	0.00	0.05
16 μM GW6471 + 19 mM DCA + 15 mM metformin	0.00	0.00	0.54	0.00	0.00	0.08

## Discussion

PGLs are rare tumors that may cause important morbidity. Currently, surgery is the only effective therapeutic option for PGLs, whereas radio- and chemotherapy give unsatisfactory responses. Thus, novel molecules for PGL treatment need to be identified.

DCA has shown a promising role as cancer treatment, since it appears more tolerated than standard cytotoxic chemotherapy^39^. In this regard, an open-label, phase 1 trial using oral DCA in patients with recurrent glioblastoma showed that chronic oral assumption of DCA is a feasible and well-tolerated drug treatment in patients with malignant gliomas^44^. Considering that DCA is a candidate therapeutic compound in some tumors and that its role in PGL is unknown, we tested its effects on unique PGL cell lines established from two patients carrying germline mutations in *SDHC* or *SDHD*, respectively. Interestingly, DCA treatment reduced viability of both PTJ64i and PTJ86i in a dose-dependent way and reduced also the growth of both PGL cell cultures. These findings are in line with those previously reported for colorectal cancer cells^33^, where DCA reduced cell growth and viability, with IC50s comparable to those observed in the present study with PGL cells. In colorectal cancer cells and in cells derived from other tumors the effects of DCA on cell viability were related to induction of apoptosis and cell cycle arrest^14,33,36,45^. A potential mechanism by which DCA affects cell growth and viability is through the increased ROS production derived from promotion of oxidative metabolism. In this regard, ROS are well known to induce cell cycle arrest and apoptosis^38,46^. In a previous study on multiple myeloma cells, the effects of DCA on cell viability were associated with PDK inhibition, promotion of oxidative phosphorylation and enhanced intracellular ROS production inducing cell cycle arrest and apoptotic cell death^36^. Also in our study the reduced viability and cell growth observed in both PGL cell lines after DCA treatment were related to restored PDH activity, reduced lactate levels and increased ROS levels, which in turn resulted in interference with cell cycle progression and induction of apoptosis. Specifically, treatment of PGL cells with DCA led to dephosphorylation of the E1α subunit of PDH complex, which is in line with the notion that DCA is able to inhibit PDK leading to PDH complex activation and promotion of oxidative metabolism, with reduction of lactate production and increase of intracellular ROS production. This resulted in cell cycle inhibition, as indicated by the altered proportion of cells in G1 and S phases, paralleled by a reduction of cell cycle protein expression. Increased ROS levels in DCA-treated PGL cells were also associated with induction of apoptosis, as shown by the increment in the percentage of Annexin-V^pos^ cells and by the increase of cytochrome C expression. In addition, DCA treatment markedly reduced clonogenic ability of both PGL cell lines, indicating that the drug had a strong and dose-dependent impact on cell self-renewal capacity. Moreover, in both cell lines DCA reduced cell migration in a dose-dependent manner. All these results indicate that DCA has a remarkable antitumor effect on PGL cell lines at dosages comparable to those employed in previous *in vitro* studies on other tumor cell lines^33,34^.

Based on these promising antitumor effects of DCA, we tested the hypothesis that combinations of this molecule with other metabolic drugs might achieve improved antitumor effects in PGL cells. In particular, we analyzed whether the combinations would be more effective on cell viability, as compared to single agents, even at lower dosages of each drug. For the combinations with DCA we selected GW6471, which we have previously shown to be active in PGL cells^9^ and metformin. The latter drug was selected because it had not been tested before in PGL and had been previously shown to have synergistic antitumor effect with DCA in other cancers^30–32,37^. In this study, we showed that metformin caused a significant reduction of PGL cell viability when used as single agent with IC50s comparable to those observed in breast and ovarian cancer cell lines^40–42^. In PTJ64i all the combinations including DCA, GW6471 and metformin caused a drastic decrease of cell viability even at the lowest drug dosages, which was much more pronounced than the reduction observed with single agents at the corresponding dosages. The effects of all these combinations were assessed by CompuSyn as deriving from synergistic or additive drug interactions. In PTJ86i the effect of the combination using the lowest drug dosages was less drastic, but even this lowest dosage combination was more effective on cell viability, as compared to the single agents at the corresponding dosages. It is worth noting that the remaining combinations with higher drug dosages had a much stronger impact on PTJ86i cell viability as compared to single agents. However, despite the stronger effects on cell viability, drug interactions were not assessed as synergistic by CompuSyn. As far as clonogenic ability, all drug combinations had drastic effects in both PTJ64i and PTJ86i cell lines. Interestingly, these effects were even more dramatic and consistent than those observed on cell viability. Moreover, in both cell lines the effects of combinations on clonogenic ability were much more pronounced as compared to single agents and all combined treatments resulted in synergistic drug interactions as assessed by CompuSyn. Overall, these findings indicate that all combinations had a strong and synergistic impact on the self-renewal capacity of the two PGL cell lines.

In conclusion, we show that DCA affects PGL cell viability by restoring PDH complex activity, increasing ROS production, interfering with cell cycle progression and promoting apoptosis. Moreover, DCA hampers cell clonogenic capacity and migration. We also show that metformin affects PGL cell viability as single agent and that the novel combination of three metabolic drugs, DCA, GW6471 and metformin has a remarkable impact on viability and self-renewal capacity of PGL cells. Our findings indicate that the three drugs used as single agents and in combination have promising antitumor effects in unique cell models of PGL.

## Materials and Methods

### Reagents and antibodies

Sodium dichloroacetate (DCA), metformin hydrochloride, 3-(4,5-Dimethyl-2-thiazolyl)-2,5-diphenyl-2H-tetrazolium bromide (MTT), crystal violet, RNAse, propidium iodide (PI), RIPA buffer and protease inhibitor cocktails were obtained from Sigma (St. Louis, MO, USA). Mouse monoclonal anti-cyclin D3 antibody was purchased from Cell Signaling Technology, Inc. (Beverly, MA, USA). Goat anti-rabbit IgG-HRP, goat anti-mouse IgG-HRP, mouse monoclonal anti-cyclin B1 and anti-cytochrome C antibodies were obtained from Santa Cruz Biotechnology, Inc. (Dallas, TX, USA). Monoclonal anti β-actin antibody was obtained from Sigma (St. Louis, MO, USA). Mouse monoclonal anti-PDH-E1α was purchased from Abcam (Cambridge MA, USA). Rabbit polyclonal PhosphoDetect^TM^ anti-PDH-E1α (pSer^300^) was purchased from Calbiochem (EMD Millipore Corp., MA, USA).

### Cells and chemicals

We have previously established and immortalized head and neck PGL cell cultures PTJ64i and PTJ86i from two patients carrying the *SDHC* c.43 C > T (p.Arg15*) and *SDHD* c.27delC (p.Val10Phefs*5) mutations, respectively^9^. PTJ64i and PTJ86i cell lines were cultured in DMEM-F12 (Sigma), supplemented with 10% FBS, at 37 °C, 5% CO_2_. Sodium dichloroacetate (DCA) and metformin were purchased from Sigma Aldrich. GW6471 was obtained from Tocris Bioscience (Bristol, UK).

### Cell viability and cell growth assays

Cell viability was tested by MTT assay. Cells were seeded in 96-well plates (4 × 10^3^ cells/well) in DMEM-F12 with 10% FBS and incubated for 24 hours before treatment. Cells were then treated for 72 hours with DCA, or metformin and GW6471 as single agents, or with combinations of the three drugs at various concentrations as indicated (5 replica wells per each condition). Subsequently, 0.5 mg/mL MTT solution was added to each well and incubated at 37 °C for at least 3 hours, until a purple precipitate was visible. In order to dissolve formazan crystals, the culture medium was replaced with dimethyl sulfoxide (DMSO, Euroclone). Absorbance of each well was quantified at 540 and 690 nm, using a Synergy H1 microplate reader (BioTek Instruments Inc., Winooski, VT, USA).

For cell counting, immortalized PGL cells were seeded in 25 cm^2^ flask (312500 cells/flask). After seeding, cells were treated with 12.5 and 50 mM DCA, or with vehicle (PBS) and counted at 24, 48 and 72 hours using the trypan blue exclusion test.

### Lactate measurement in cell culture medium

Lactate levels in growth media of PGL cells treated and untreated with DCA were measured as previously described^47^. Briefly, cells were seeded in 12-well plates (1 × 10^5^ cells/well) and, following cell attachment, they were treated for 24 hours with 50 mM DCA, or with vehicle. Then, culture supernatants were collected, clarified by centrifugation and analyzed for lactate levels using a Lactate Pro Analyser (Arkray Inc. Kyoto, Japan).

### Detection of reactive oxygen species (ROS)

ROS production was determined by monitoring the increase of green fluorescence of CM-H2DCFDA, an indicator for ROS in cells. Fluorescence was monitored by the FACSCanto flow cytometer with the FL1 detector in a log mode using the FACSDiva analysis software (both from BD Biosciences), after labelling cells (3 × 10^5^ cells/T25 flask) with 5 µM of CM-H2DCFDA (Life Technologies, Milano, Italy) for 1 hour at 37 °C. The mean fluorescence intensity (MFI) was obtained by histogram statistics and utilized to quantify ROS production. For each sample, 2 × 10^4^ events were analyzed.

### Cell cycle analysis

Approximately 0.5 × 10^6^ cells per experimental condition were harvested, fixed in 70% cold ethanol and kept at 4 °C overnight. Cells were then resuspended in 20 µg/ml PI and 100 µg/ml RNAse, final concentrations. Cell cycle profiles (10^5^cells) were analyzed by a CytoFLEX flow cytometer with the FL3 detector in a linear mode using the CytoExpert analysis software (both from Beckmann Coulter, Milano, Italy). Data were analyzed with ModFit software (Verity Software House, ME, USA).

### Apoptosis assay

To assess apoptosis, a commercial Annexin-V-FITC/PI Kit (Bender Med System, Vienna, Austria) was used according to the manufacturer’s instructions. Briefly, the cells were gently resuspended in binding buffer and incubated with Annexin-V-FITC for 10 min at room temperature in the dark. Samples were then washed and analyses were performed with a FACSCanto flow cytometer with the FL1 detector in a log mode using the FACSDiva analysis software (both from BD Biosciences, Milano, Italy). For each sample, at least 10^5^ events were collected. Viable cells are Annexin-V^neg^ while apoptotic cells are Annexin-V^pos^.

### Western blotting analysis

PGL cell lines treated with 12.5 and 50 mM DCA, or with vehicle for 24 hours, were lysed in RIPA buffer containing protease inhibitors cocktails and phosphatase inhibitors. The lysates were quantified by the BCA Protein Assay (Thermo Scientific, Rockford, IL, USA) and 30 μg were subjected to electrophoresis followed by immunoblotting. The membranes were blocked in 5% nonfat dry milk for one hour at room temperature and incubated with the appropriate primary antibodies. Then the membranes were incubated with either anti-rabbit or anti-mouse (1:3000) HRP-conjugated secondary antibodies. The blots were revealed by chemiluminescence using the SuperSignal West Pico Chemiluminescence Substrate (Thermo Scientific, Rockford, IL, USA) according to the manufacturer’s instructions. Analysis of β-actin was performed as loading control.

### Clonogenic assay

PGL cells (200 cells/well) were seeded into 6-well plates and following cell attachment they were treated for 72 hours with single agents, or combinations as indicated. Then, after medium refreshment, the plates were incubated at 37 °C with 5% CO_2_, until cells in the control vehicle formed colonies consisting of at least 50 cells (12–15 days). Colonies were fixed with 75% methanol and stained with 0.5% crystal violet, then rinsed with tap water, dried and counted.

### *In vitro* wound-healing assay

PGL cells were seeded in 12-well plates (2 × 10^5^ cells/well) and incubated in medium containing 10% FBS. After the cells grew to confluence, they were incubated overnight in serum-free medium and wounds were made by sterile pipette tips. Plates were washed twice with PBS in order to remove the detached cells and were incubated with serum-free medium containing 12.5 or 50 mM DCA, or with vehicle. Pictures were taken at 0 h (immediately after scratching) and at the indicated time intervals, until the wound closure was completed by cells treated with vehicle. The number of cells migrated from the wound edge into the uncovered area was quantified in three fields per well. Data were expressed as ratio of migrated cells after DCA treatment *versus* vehicle.

### IC50 and combination index (CI) calculation

IC50 values were calculated using the CompuSyn software. The interaction among DCA, GW6471 and metformin was quantified by determining the combination index (CI). The CI was calculated by CompuSyn software using the Chou-Talalay equation^39^. Based on this equation CI < 1 indicates synergistic effects, CI = 1 indicates additive effects and CI > 1 indicates antagonistic effects.

### Statistical analysis

Statistical analyses were performed by the independent samples t-test using the Dunnett’s test for multiple comparisons where appropriate. A *p*-value of 0.05 was considered statistically significant.

## Electronic supplementary material

Supplementary Information

## Data Availability

All data underlying the findings described within the manuscript are fully available without restrictions.
