# Supplementary material for: Effects of dichloroacetate as single agent or in combination with GW6471 and metformin in paraganglioma cells
Source: Sci Rep. 2018 Sep 11;8:13610. doi: 10.1038/s41598-018-31797-5 (PMC6134030; doi:10.1038/s41598-018-31797-5)

SREP-18-02002A

Supplementary Information.

Effects of dichloroacetate as single agent or in combination with GW6471 and metformin in paraganglioma cells

Rosalba Florio[1,2], Laura De Lellis[1,2*], Serena Veschi[1], Fabio Verginelli[1,2], Viviana di Giacomo[1], Marialucia Gallorini[1], Silvia Perconti[2], Mario Sanna[4], Renato Mariani Costantini[2,3], Angelica Natale[1], Arduino Arduini[5], Rosa Amoroso[1], Amelia Cataldi[1], Alessandro Cama[1,2*].

[1]Department of Pharmacy, "G. d'Annunzio" University of Chieti-Pescara, Chieti, Italy; [2]Unit of General Pathology, CeSI-MeT, University of Chieti, Chieti (Italy); [3]Department of Medical, Oral and Biotechnological Sciences, "G. d'Annunzio" University of Chieti-Pescara, Chieti, Italy; [4]Department of Otology and Skull Base Surgery, Gruppo Otologico, Piacenza, Italy; [5]R&D Department, CoreQuest Sagl, Manno, Switzerland.

INDEX PAGE

INDEX	PAGE
Supplementary Table S1	S3
Supplementary Table S2	S4
Supplementary Figure S1 (full-length blots of Figure 2)	S5
Supplementary Figure S2 (full-length blots of Figure 3)	S6
Supplementary Figure S3 (full-length blots of Figure 4)	S7

Table S1. Statistical analysis of the effects of DCA, GW6471 and metformin as single agents versus drug combinations on the viability of PTJ64i and PTJ86i cell lines.

PTJ64i

		**7 μM GW**	**16 μM GW**	**10 mM DCA**	**19 mM DCA**	**6 mM metformin**	**15 mM metformin**
	Cell viability % (± SD)	98.29 (± 9.16)	39.58 (± 10.97)	69.66 (± 2.91)	25.06 (± 0.78)	81.20 (± 2.59)	52.73 (± 9.61)
**7 μM GW + 10 mM DCA + 6 mM metformin**	9.94 (± 4.38)	p=0.0001***		p<0.0001***		p<0.0001***	
**16 μM GW + 10 mM DCA + 6 mM metformin**	3.36 (± 0.81)		p=0.0047**	p<0.0001***		p<0.0001***	
**16 μM GW + 10 mM DCA + 15 mM metformin**	2.65 (± 0.35)		p=0.0043**	p<0.0001***			p=0.0008***
**16 μM GW + 19 mM DCA + 15 mM metformin**	1.81 (± 0.46)		p=0.0040**		p<0.0001***		p=0.0008***

Statistical analysis was performed by unpaired Student's t-test (***p* <0.01; ****p* <0.001).
Data shown are the means ± standard deviation (SD) of three independent experiments.

PTJ86i

		**7 μM GW**	**16 μM GW**	**10 mM DCA**	**19 mM DCA**	**6 mM metformin**	**15 mM metformin**
	Cell viability % (± SD)	98.51 (± 5.71)	63.82 (± 3.20)	86.58 (± 11.91)	50.49 (± 6.87)	95.04 (± 2.06)	74.95 (± 1.86)
**7 μM GW + 10 mM DCA + 6 mM metformin**	51.49 (± 4.43)	p=0.0004***		p=0.0088**		p=0.0001***	
**16 μM GW + 10 mM DCA + 6 mM metformin**	18.71 (± 4.62)		p=0.0002***	p=0.0008***		p<0.0001***	
**16 μM GW + 10 mM DCA + 15 mM metformin**	16.25 (± 3.27)		p<0.0001***	p=0.0006***			p<0.0001***
**16 μM GW + 19 mM DCA + 15 mM metformin**	5.14 (± 3.31)		p<0.0001***		p=0.0005***		p<0.0001***

Statistical analysis was performed by unpaired Student's t-test (***p* <0.01; ****p* <0.001)
Data shown are the means ± standard deviation (SD) of three independent experiments.

Table S2. Statistical analysis of the effects of DCA, GW6471 and metformin as single agents versus drug combinations on the clonogenicity of PTJ64i and PTJ86i cell lines.

		PTJ64i					
		**7 µM GW**	**16 µM GW**	**10 mM DCA**	**19 mM DCA**	**6 mM metformin**	**15 mM metformin**
	# of colonies (± SD)	95.0 (± 7.07)	40.0 (± 0.00)	69.5 (± 6.36)	21.0 (± 1.14)	80.5 (± 9.19)	52.5 (± 6.36)
**7 µM GW + 10 mM DCA + 6 mM metformin**	4.50 (± 0.71)	p=0.0031**		p=0.0048**		p=0.0073**	
**16 µM GW + 10 mM DCA + 6 mM metformin**	0.50 (± 0.71)		p=0.0002***	p=0.0043**		p=0.0066**	
**16 µM GW + 10 mM DCA + 15 mM metformin**	0.0 (± 0.00)		p<0.0001***	p=0.0042**			p=0.0073**
**16 µM GW + 19 mM DCA + 15 mM metformin**	0.0 (± 0.00)		p<0.0001***		p=0.0023**		p=0.0073**

Statistical analysis was performed by unpaired Student's t-test (***p* <0.01; ****p*<0.001).
Data shown are the means ± standard deviation (SD) of two independent experiments.

		PTJ86i					
		**7 µM GW**	**16 µM GW**	**10 mM DCA**	**19 mM DCA**	**6 mM metformin**	**15 mM metformin**
	# of colonies (± SD)	59.0 (± 1.41)	41.5 (± 2.12)	59.5 (± 2.12)	30.5 (± 7.78)	69.0 (± 1.41)	62.0 (± 4.24)
**7 µM GW + 10 mM DCA + 6 mM metformin**	10.5 (± 2.12)	p=0.0014**		p=0.0019**		p=0.0009***	
**16 µM GW + 10 mM DCA + 6 mM metformin**	0.0 (± 0.00)		p=0.0013**	p=0.0006***		p=0.0002***	
**16 µM GW + 10 mM DCA + 15 mM metformin**	0.0 (± 0.00)		p=0.0013**	p=0.0006***			p=0.0023**
**16 µM GW + 19 mM DCA + 15 mM metformin**	0.0 (± 0.00)		p=0.0013**		p=0.0310*		p=0.0023**

Statistical analysis was performed by unpaired Student's t-test (**p* <0.05; ***p*<0.01; ****p*<0.001).
Data shown are the means ± standard deviation (SD) of two independent experiments.

Supplementary Figure S1. Full-length western blots of p-PDH-E1α (pSer^{300}), PDH-E1α and β-actin in PTJ64i and PTJ86i cell lines, treated or not with 12.5 or 50 mM DCA (the corresponding cropped blots are shown in Figure 2 of the main text). The full-length membrane was cut, incubated with the first antibody, then stripped and reprobed with the next antibody.

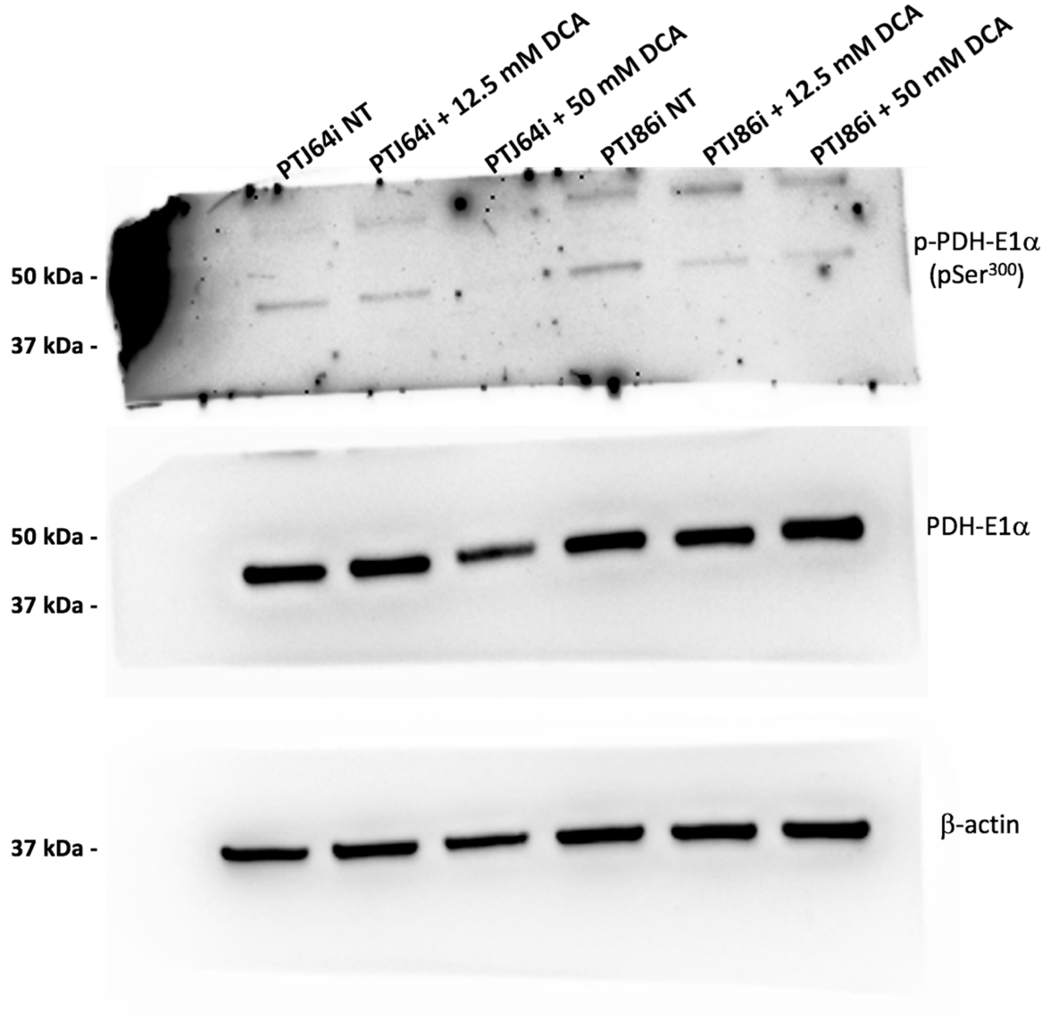

Supplementary Figure S2. Full-length western blots of cyclin B1, cyclin D3 and β-actin in PTJ64i and PTJ86i cell lines, treated or not with 50 mM DCA (the corresponding cropped blots are shown in Figure 3 of the main text). The full-length membrane was incubated with the first antibody, then stripped and reprobed with the next antibody.

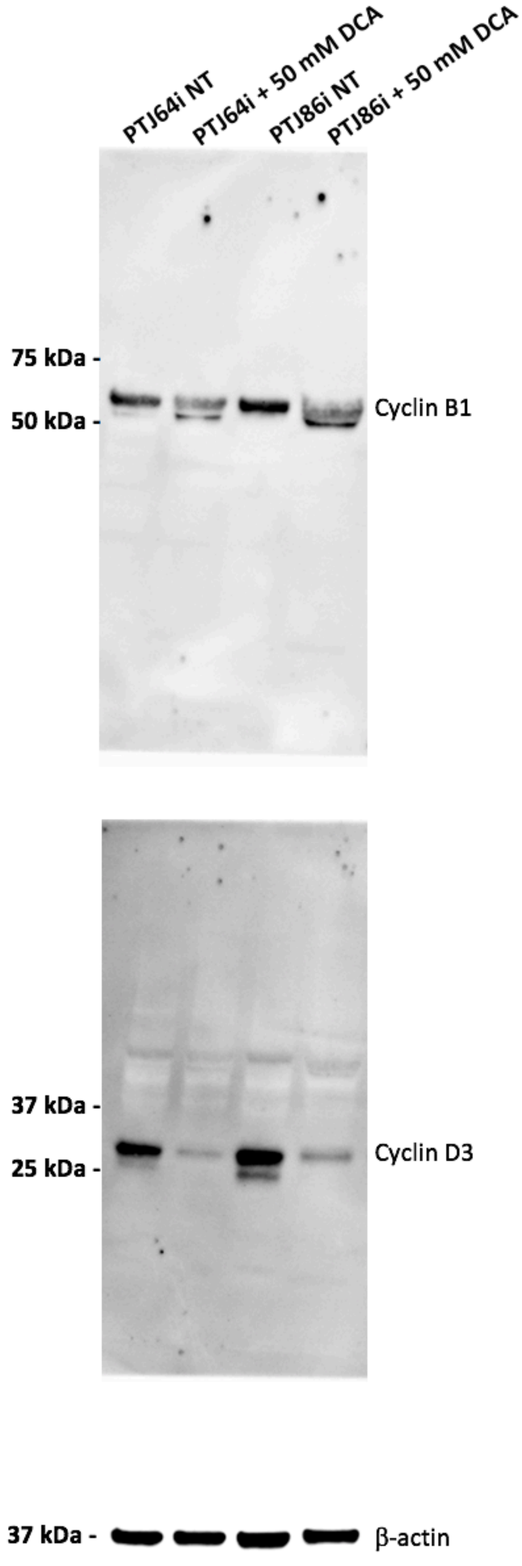

Supplementary Figure S3. Full-length western blots of cytochrome C and β-actin in PTJ64i and PTJ86i cell lines, treated or not with 12.5 or 50 mM DCA (the corresponding cropped blots are shown in Figure 4 of the main text). The full-length membranes were cut and incubated with the indicated antibodies.

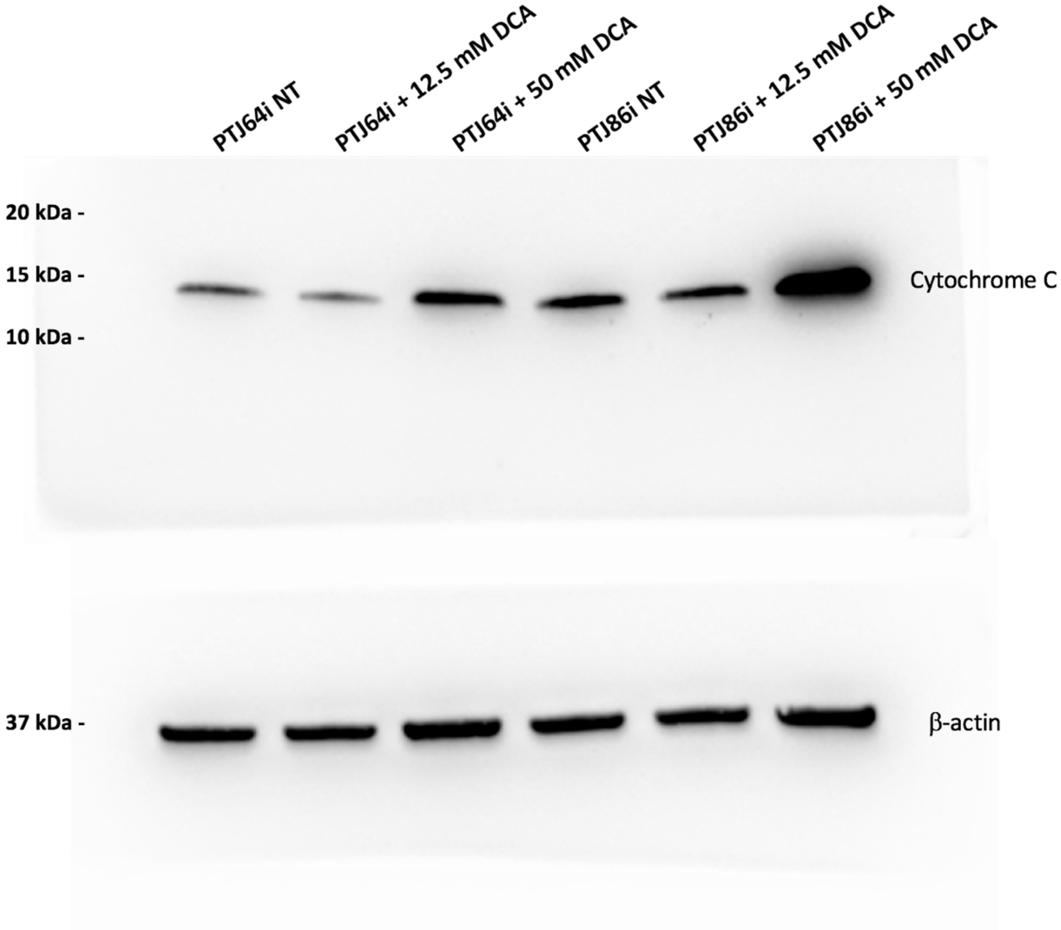

Supplement: Supplementary file 1 — Supplementary Information [file 41598_2018_31797_MOESM1_ESM.pdf]
